# Preclinical magnetic resonance imaging of proinflammatory epicardial adipose tissue: accelerated methods for simultaneous fatty acid composition and relaxation parameter mapping with relationships to tissue biomarkers

**DOI:** 10.1016/j.jocmr.2025.101947

**Published:** 2025-08-22

**Authors:** Julia E. Bresticker, Caitlin M. Pavelec, John T. Echols, Amit R. Patel, Matthew J. Wolf, Frederick H. Epstein

**Affiliations:** a Biomedical Engineering, University of Virginia, Charlottesville, Virginia, USA; b Division of Cardiovascular Medicine, University of Virginia, Charlottesville, Virginia, USA; c Robert M. Berne Cardiovascular Research Center, University of Virginia, Charlottesville, Virginia, USA; d Radiology, University of Virginia, Charlottesville, Virginia, USA

**Keywords:** Epicardial adipose tissue, Fatty acid composition, T1 mapping, Preclinical imaging

## Abstract

**Background:**

Epicardial adipose tissue (EAT) plays a central role in metabolic heart disease through local inflammatory signaling. In obesity, EAT undergoes pathological remodeling marked by increased adipocyte size, saturated fatty acids (SFAs), macrophage infiltration, and inflammatory cytokine secretion. Proton density fat fraction (PDFF), relaxation times, and the fatty acid composition (FAC) (the amount of SFAs, monounsaturated fatty acids [MUFAs], and polyunsaturated fatty acids [PUFAs]) are promising metrics of EAT quality, yet their role as biomarkers of proinflammatory EAT has not been established. This study presents an accelerated cardiovascular magnetic resonance (CMR) method for simultaneous EAT FAC and relaxation time mapping and evaluates their relationships with histological and cytokine markers of inflammation.

**Methods:**

An electrocardiogram (ECG)-gated inversion recovery multi-echo gradient-echo sequence with radial golden-angle sampling was developed for simultaneous FAC and relaxation time mapping. A high-dimensionality patch-based low-rank reconstruction was applied to undersampled images. Phantom validation was performed using oil mixture and gadolinium phantoms, followed by in vivo imaging of mice (n = 16–20/group) fed a high-fat high-sucrose diet (HFHSD), HFHSD plus the sodium-glucose cotransporter-2 inhibitor (SGLT2i) empagliflozin (HFHSD+EMPA), or a high-fat diet (HFD). PDFF, SFA fraction, MUFA fraction, PUFA fraction, R_2_*, and T_1_ measurements were made in EAT and subcutaneous adipose tissue (SAT). EAT FAC values were indexed to those of SAT. Ex vivo histology and cytokine assays were used to assess EAT and myocardial inflammation.

**Results:**

Phantom validation demonstrated strong agreement between CMR-derived and reference FAC and T_1_ values (r>0.94, p<0.05). Diet-induced changes in adipose tissue FAC were detected by CMR. HFHSD+EMPA mice had lower EAT SFA index than both HFHSD (p<0.01) and HFD (p<0.05) mice, and higher MUFA index (p<0.01), PUFA index (p<0.05), and T_1_ (p<0.05) compared HFHSD mice. EAT SFA index positively correlated with macrophage infiltration and proinflammatory cytokines, while MUFA and PUFA indexes were inversely correlated with proinflammatory cytokines. EAT T_1_ negatively correlated with adipocyte hypertrophy.

**Conclusion:**

This study developed an accelerated EAT FAC and relaxation time mapping method and provides evidence that MRI-derived EAT FAC indexes and relaxation times may serve as biomarkers of proinflammatory EAT by demonstrating correlations with histological and cytokine markers.

## Graphical abstract

**Figure:**
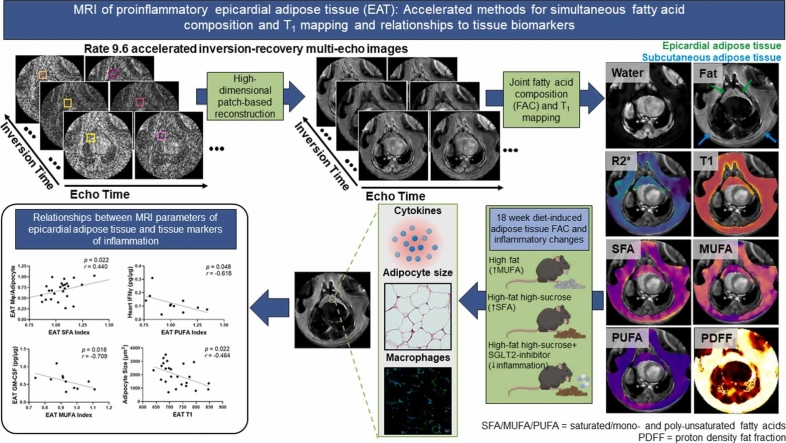

## 1 Background

Epicardial adipose tissue (EAT) is a metabolically active visceral fat depot surrounding the heart which has emerged as a key player in the pathophysiology of various heart diseases, including atrial fibrillation, coronary artery disease, coronary microvascular disease, and heart failure with preserved ejection fraction (HFpEF) [1], [2], [3]. Notably, recent studies have shown that increased visceral adiposity is a near-universal feature of HFpEF, highlighting its integral role in disease pathophysiology [4]. Unlike other visceral fat depots, EAT is in direct contact with the myocardium and coronary arteries, without a fascia or other physical barrier to separate it from the heart. This unique anatomical feature allows for direct crosstalk between EAT and the myocardium through a shared microcirculation [5]. In healthy conditions, EAT supports cardiac function by providing fatty acids as an energy source, buffering excess circulating lipids, and maintaining features of brown and beige adipose tissue that support thermogenesis and cardiometabolic health [6]. However, in obesity and metabolic disease, EAT undergoes pathological remodeling, characterized by adipocyte hypertrophy, oxidative stress, and a shift in fatty acid composition (FAC) toward a proinflammatory profile [7], [8], [9]. Specifically, EAT in obesity becomes enriched with saturated fatty acids (SFAs) which activate toll-like receptor 4 signaling and the NLRP3 inflammasome [10], [11]. This cascade promotes macrophage recruitment, polarization toward a proinflammatory M1 phenotype, and secretion of proinflammatory cytokines into the coronary microcirculation [12]. In contrast, monounsaturated (MUFAs) and polyunsaturated fatty acids (PUFAs) have been shown to impede NLRP3 inflammasome activity and mitigate inflammation, highlighting the FAC as a key mediator of EAT-driven cardiometabolic dysfunction [7], [11]. Given the role of proinflammatory EAT in cardiovascular disease, it is emerging as a target for therapies such as sodium-glucose cotransporter-2 inhibitors (SGLT2i), which significantly improve cardiovascular outcomes in HFpEF [13], [14]. These drugs may exert their cardioprotective effects in part by modulating EAT biology, decreasing macrophage infiltration and promoting adipose tissue browning [15]. In this context, noninvasive imaging to assess proinflammatory EAT would have many potential applications.

MRI-based assessment of EAT has primarily focused on volume quantification, yet volume alone may not best reflect its proinflammatory state. Beyond EAT quantity, MRI techniques such as proton density fat fraction (PDFF) and FAC mapping enable the evaluation of adipose tissue quality. Prior studies using MRI FAC methods have shown that EAT SFA fraction is associated with left ventricular structural and functional impairments [16] as well as coronary microvascular dysfunction [17], suggesting their potential as biomarkers of proinflammatory EAT. Separately, T_1_ mapping has shown promise in detecting adipose tissue remodeling, with longer T_1_ relaxation times observed in adipose tissue from healthy individuals compared to those with obesity and in visceral adipose tissue compared to subcutaneous adipose tissue (SAT) [18]. While EAT FAC MRI techniques have been applied in both preclinical [17] and clinical [16] settings, EAT T_1_ mapping remains largely unexplored, and a joint approach to estimate both EAT FAC and T_1_ has yet to be developed. Currently, FAC and T_1_ mapping utilize separate acquisitions, which is time-inefficient. A joint approach to EAT FAC and T_1_ mapping could offer a time-efficient and comprehensive characterization of EAT beyond volume alone.

While CMR-derived biomarkers for the assessment of EAT quality show promise, their relationships with direct histological and molecular tissue biomarkers remain largely unexamined. Establishing these relationships is essential for validating CMR as a noninvasive tool to assess proinflammatory EAT and its role in heart disease. In particular, showing how CMR mapping parameters relate to adipocyte morphology, macrophage infiltration, and cytokine expression would establish a foundation for using MRI biomarkers for risk stratification and treatment monitoring.

This study presents an accelerated CMR method for simultaneous FAC and relaxation time mapping of EAT in mice at 9.4T, enabling a more comprehensive assessment of its proinflammatory phenotype. Applying this novel method to diet-induced mouse models of metabolic heart disease, we investigate relationships between CMR-derived EAT parameters and ex vivo tissue measurements of inflammation, including macrophage presence, adipocyte morphology, and cytokine expression. Through these studies, we aim to establish CMR-based biomarkers for the noninvasive assessment of proinflammatory EAT.

## 2 Theory

### 2.1 Signal model for joint FAC and relaxation time mapping

The mean triglyceride spectrum can be characterized by nine distinct hydrogen-1 (^1^H) resonances (j∈{A,…,I}) with relative magnitudes $\rho_{j}$ and chemical shifts $\delta_{j}$ relative to the water proton resonance $\delta_{w}$ (Table 1) [19]. The relative phase of each resonance at time, t, after excitation is given by $\alpha_{j}\left(t\right)=\;e^{i\gamma B_{0}\left(\delta_{j}-\delta_{w}\right)t}$, where $B_{0}$ is the main magnetic field strength and γ is the gyromagnetic ratio.

**Table 1:** Triglyceride ^1^H resonances and their corresponding chemical shifts (δ) and relative magnitudes (ρ). cl, chain length; ndb, number of double bonds; nmidb, number of methylene-interrupted double bonds.

Resonance	Type	δ [ppm]	Relative magnitude (ρ)
A	Methyl	0.90	9
B	Methylene	1.30	$\left[\left(\mathrm{cl}-4\right)\times 6\right]-\left(\mathrm{ndb}\times 8\right)+(\mathrm{nmidb}\times 2)$
C	β-Carboxyl	1.60	6
D	α-Olefinic	2.02	$\left(\mathrm{ndb}-\mathrm{nmidb}\right)\times 4$
E	α-Carboxyl	2.24	6
F	Diacyl	2.75	nmidb×2
G	Glycerol	4.20	4
H	Glycerol	5.19	1
I	Olefinic	5.29	ndb×2

Water (W) and fat (F) components along with the $T_{1}$ relaxation time can be determined from a set of inversion recovery gradient-echo images acquired at multiple inversion times (${\mathrm{TI}}_{m}$, m=1,…,M) and echo times (${\mathrm{TE}}_{n}$, n=1,…,N). The complex MR signal y at a given voxel is expressed as:

(1) yTEn,TIm=W+F∑j=19ρjαjTEnei2πψTEneiϕe−R2*TEnA0−Bdiffe-TImT1*

Here, ψ represents off-resonance frequency due to static field inhomogeneity, ϕ is the initial phase, $R_{2}^{*}$ is the transverse relaxation rate, and $T_{1}^{*}$ is the apparent longitudinal relaxation time that differs from the true T_1_ due to radiofrequency pulse effects on signal recovery. The constants, $A_{0}$ and $B_{\mathrm{diff}}$ correspond to the equilibrium signal and the signal difference between the initial (post-inversion) and equilibrium states. The true T_1_ can be approximated using the conventional Look-Locker correction [20]:

(2) $$\begin{array}{c} T_{1}=T_{1}^{*}\left(\frac{B_{\mathrm{diff}}}{A_{0}}-1\right) \end{array}$$

### 2.2 Characterization of triglyceride saturation

The relative magnitude of each triglyceride resonance is determined by the (1) number of -CH=CH- double bonds per triglyceride (ndb), (2) number of methylene- interrupted double bonds (nmidb), and (3) the fatty acid chain length (cl) (Table 1) [19]. To reduce the number of unknown parameters, the average chain length can be estimated based on prior knowledge. These parameters allow decomposition of the fat signal, F, into distinct triglyceride subcomponents:

(3) $$\begin{array}{c} F=F_{\mathrm{ntg}}+F_{\mathrm{ndb}}+F_{\mathrm{nmidb}} \end{array}$$

where $F_{\mathrm{ntg}}$ is proportional to the number of triglycerides, $F_{\mathrm{ndb}}$ to the number of double bonds, and $F_{\mathrm{nmidb}}$ to the number of methylene-interrupted double bonds.

The absolute values of ndb and nmidb per “mean triglyceride” within a voxel are calculated as:

(4) $$\begin{array}{c} \mathrm{ndb}=\;\frac{F_{\mathrm{ndb}}}{F_{\mathrm{ntg}}}\; \end{array}$$

(5) $$\begin{array}{c} \mathrm{nmidb}=\;\frac{F_{\mathrm{nmidb}}}{F_{\mathrm{ntg}}}.\; \end{array}$$

The relative fractions of unsaturated fatty acids (UFAs) and PUFAs are given by:

(6) $$\begin{array}{c} \text{UFA }=\frac{\mathrm{ndb}-\mathrm{nmidb}}{3} \end{array}$$

(7) $$\begin{array}{c} \text{PUFA }=\frac{\mathrm{nmidb}}{3}.\; \end{array}$$

Relative amounts of SFAs and MUFAs are determined using the relationships UFA = MUFA + PUFA and UFA + SFA = 100% [21].

### 2.3 Least squares approximation

The complex signal for N echo times and M inversion times $y={\left[y\left(TE_{1},TI_{1}\right),\;\ldots ,y\left(TE_{N},TI_{1}\right),\;\ldots ,y\left(TE_{1},TI_{M}\right),\;\ldots ,y\left(TE_{N},TI_{M}\right)\right]}^{T}$ can be expressed in matrix form as:

(8) $$\begin{array}{c} y=\Psi \mathrm{Ax}e^{-i\phi },\; \end{array}$$

where

(9) $$\begin{array}{c} A_{\left(N*M\right)\times 4}=\left[\begin{array}{c} \begin{array}{c} \alpha_{1,1,W}\\ \vdots \\ \alpha_{N,1,W} \end{array}\begin{array}{ccc} \alpha_{1,1,A} & \ldots & \alpha_{1,1,I}\\ \vdots & \ddots & \vdots \\ \alpha_{N,1,A} & \ldots & \alpha_{N,1,I} \end{array}\\ \begin{array}{c} \alpha_{1,2,W}\\ \vdots \\ \alpha_{N,M,W} \end{array}\begin{array}{ccc} \alpha_{1,2,A} & \ldots & \alpha_{1,2,I}\\ \vdots & \ddots & \vdots \\ \alpha_{N,M,A} & \ldots & \alpha_{N,M,I} \end{array} \end{array}\right]\times \begin{array}{c} \underbrace{\left[\begin{array}{cccc} 1 & 0 & 0 & 0\\ 0 & 9 & 0 & 0\\ 0 & 6(cl-4) & -8 & 2\\ 0 & 6 & 0 & 0\\ 0 & 0 & 4 & -4\\ 0 & 6 & 0 & 0\\ 0 & 0 & 0 & 2\\ 0 & 4 & 0 & 0\\ 0 & 1 & 0 & 0\\ 0 & 0 & 2 & 0 \end{array}\right]}\\ \rho \end{array}.\; \end{array}$$

Here, $x={\left[W,\;F_{\mathrm{ntg}},F_{\mathrm{ndb}},\;F_{\mathrm{nmidb}}\right]}^{T}$ is the unknown parameter vector, and Ψ is the (N*M)×(N*M) block-diagonal matrix:

(10) $$\begin{array}{c} \Psi =\text{blkdiag}\underset{M\text{ times}}{\underset{︸}{\left(D,D,\ldots D\right)}},\; \end{array}$$

where each N×N diagonal block is

(11) $$\begin{array}{c} D= \end{array}\left(\begin{array}{cccc} e^{i2\pi \psi TE_{1}} & 0 & \ldots & 0\\ 0 & e^{i2\pi \psi TE_{2}} & \ldots & 0\\ \vdots & \vdots & \ddots & \vdots \\ 0 & 0 & \ldots & e^{i2\pi \psi TE_{N}} \end{array}\right).$$

The ρ matrix describes the relative weights of water and ^1^H triglyceride resonances as a function of the average fatty acid chain length, which is set to 16.12 based on prior knowledge of the visceral adipose tissue lipidome of mice fed a high-fat high-sucrose diet [22]. The terms

(12) αn,m,W=A0−Bdiffe-TImT1*e−R2*TEn

and

(13) αn,m,j=A0−Bdiffe-TImT1*eiγB0δj−δwTEne−R2*TEn

account for transverse and longitudinal relaxation effects and chemical shift-induced frequency changes of water and the $j^{\mathrm{th}}$ fat resonance.

The objective is to estimate the unknown parameter $T_{1}^{*}$ and the vector x containing the water and fat components along with the confounding parameters ψ, ϕ, $R_{2}^{*}$, $A_{0}$, and $B_{\mathrm{diff}}$. The longitudinal relaxation parameters $A_{0}$, $B_{\mathrm{diff}}$, and $T_{1}^{*}$ are estimated by minimizing the squared residual error between the signal at $TE_{1}$, $y_{TE_{1}}=[y\left(TE_{1},TI_{1}\right),\;\ldots ,y\left(TE_{1},TI_{M}\right)]$, and the three-parameter model $\left(A_{0}-B_{\mathrm{diff}}e^{-\mathrm{TI}/T_{1}^{*}}\right)$ using the Levenberg-Marquart algorithm. Once $A_{0}$, $B_{\mathrm{diff}}$, and $T_{1}^{*}$ are determined, the separable nonlinear problem can be solved using variable projection [21].

The confounding parameters ψ, ϕ, and $R_{2}^{*}$ are estimated by minimizing

(14) JR2*,ψ,ϕ=y−ΨAReΑHA−1ReΑHΨHye−iϕeiϕ2.

The phase term is determined analytically as

(15) ϕˆ=12argAHΨHyTReAHA−1(AHΨHy)

for a given $R_{2}^{*}$ and ψ.

### 2.4 Multicontrast image reconstruction

Images acquired at multiple TEs and TIs can be modeled as a superposition of a limited number of spectral components. Assuming that nonlinear variations vary smoothly over small regions, local image patches can be approximated as spectrally sparse, with signal primarily arising from modeled water and fat components. These properties support the use of higher-order low-rank regularization to exploit spatial similarity and redundancy through contrast dimensions.

A high-dimensionality undersampled patch-based reconstruction (HD-PROST) framework was employed to jointly enforce data consistency and a local low-rank structure [23]. The objective is to recover denoised images $X\in C^{n_{x}\times n_{y}\times N\times M}$ of size $n_{x}\times n_{y}$ with N TEs and M TIs from undersampled radial k-space data Y. Assuming that X can be represented as a higher-order low-rank tensor on a patch scale, the reconstruction problem is formulated as:

(16) $$\begin{array}{c} \underset{X}{\mathrm{argmin}}\frac{1}{2}{\left\|EX-Y\right\|}_{F}^{2}+\sum_{p}\lambda_{p}{\left\|T_{p}\right\|}_{*}\;s.t.\;T_{p}=P_{p}\left(X\right) \end{array}$$

Here, E=FE, where F is the nonuniform fast Fourier transform (NUFFT) [24] and E is the ESPIRiT operator [25]. The operator $P_{p}(X)$ extracts a patch of size P centered at pixel p, with patches stacked along the contrast dimensions to form a tensor $T_{p}\in C^{P\times N\times M}$. The nuclear norm ${\left\|T_{p}\right\|}_{*}$ and regularization parameter $\lambda_{p}$ promote low-rank across spatial, TE, and TI dimensions.

**Fig. 1:**
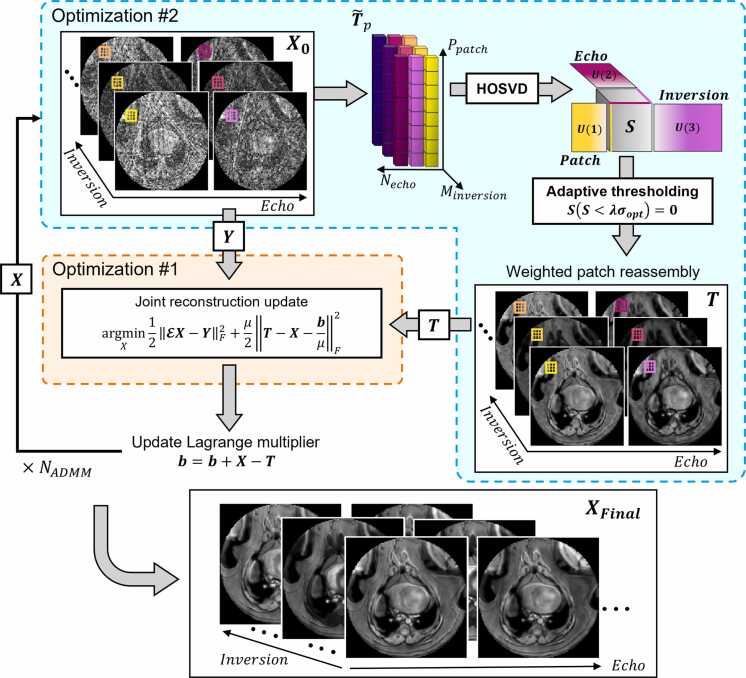
Image reconstruction framework. The HD-PROST-based reconstruction alternates between two subproblems to recover denoised multicontrast images from undersampled radial k-space data. Optimization 2: The initial image estimate $X_{0}$, generated via NUFFT, is divided into overlapping patches. Patches from the same spatial location across all contrast dimensions are unfolded into 3D tensors (${\tilde{T}}_{p}$) and denoised using HOSVD followed by adaptive hard thresholding. Denoised patches are reassembled into a tensor T. Optimization 1: A joint reconstruction update enforces data consistency with the measured k-space data Y and regularization toward the denoised image T, yielding an updated image X. The Lagrange multiplier (b) is updated after each iteration. This process is repeated for $N_{\mathrm{ADMM}}$ iterations to produce the final reconstructed image series $X_{\mathrm{Final}}$. $\sigma_{\mathrm{opt}}$ optimal hard threshold. *HOSVD* higher-order singular value decomposition, *NUFFT* nonuniform fast Fourier transform, *3D* three dimensional, *HD-PROST* high-dimensionality undersampled patch-based reconstruction

This problem is solved using the alternating direction method of multipliers (ADMM), which decomposes the problem into two alternating subproblems [26]. This process is outlined in Fig. 1.

#### 2.4.1 Optimization 1: joint reconstruction

Given a denoised tensor T from Optimization 2 (as shown in Fig. 1), the image X is updated by solving a Tikhonov-regularized least squares problem to enforce consistency with the measured k-space data:

(17) $$\begin{array}{c} \underset{X}{\mathrm{argmin}}\frac{1}{2}{\left\|EX-Y\right\|}_{F}^{2}+\frac{\mu }{2}{\left\|T-X-\frac{b}{\mu }\right\|}_{F}^{2} \end{array}\;$$

where μ is a penalty parameter and b is the augmented Lagrange multiplier. The problem is solved using the conjugate gradient algorithm. After each iteration, the Lagrange multiplier is updated as $b^{\left(i\right)}=b^{\left(i-1\right)}+X^{\left(i\right)}-T^{\left(i\right)}$.

#### 2.4.2 Optimization 2: local low-rank denoising

The current image estimate X is divided into overlapping patches, each reshaped into a tensor ${\tilde{T}}_{p}\in C^{P\times N\times M}$. Due to spatial similarity and redundancy across contrast dimensions, each tensor is expected to exhibit low-rank structure and is denoised using higher-order singular value decomposition (HOSVD). Orthonormal basis matrices $U_{\left(1\right)}$, $U_{\left(2\right)},\;$and $U_{\left(3\right)}$ are computed for the spatial, TE, and TI modes, respectively, and used to compute the core tensor:

(18) $$\begin{array}{c} S_{P}={\tilde{T}}_{P}\times_{1}U_{\left(1\right)}^{H}\times_{2}U_{\left(2\right)}^{H}\times_{3}U_{\left(3\right)}^{H} \end{array}$$

where $\times_{i}$ represents the $i^{\mathrm{th}}$ mode product.

Singular value hard thresholding is applied to the core tensor, where components below an adaptive threshold are removed as they are assumed to primarily represent noise. Following the method proposed by Gavish and Donoho, the optimal hard threshold for a given patch core tensor $\lambda_{p}$ is set to 2.39 times the median singular value across all dimensions [27]. The denoised patch tensor is then reconstructed by multiplying the thresholded core tensor with the corresponding orthonormal bases along each mode. All denoised patches are combined using weighted averaging of overlapping voxels, yielding the tensor T, which is used as the reference image in Optimization 1.

## 3 Methods

### 3.1 Pulse sequence

**Fig. 2:**
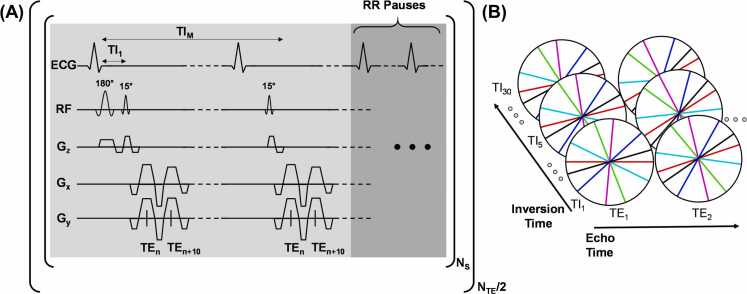
Pulse sequence diagram. (A) Inversion recovery interleaved multi-echo gradient-echo pulse sequence for M TIs and N TEs, showing the acquisition of a single radial spoke at TE_n_ and TE_n+10_ across multiple TIs, followed by RR pauses. (B) Example k-space trajectories with golden-angle rotation in-plane and through contrast dimensions, illustrating six spokes acquired at different TEs and TIs. *N*_*s*_ number of spokes, *N_TE_* number of total echo times, *TI* inversion times, *TE* echo times

The proposed joint FAC and T_1_ mapping pulse sequence is shown in Fig. 2A. An electrocardiogram (ECG)-gated inversion recovery prepared interleaved multi-echo gradient-echo pulse sequence with 2D radial trajectories was developed to obtain images at multiple TEs and TIs. A slab-selective adiabatic 180° pulse was applied prior to initial excitation. Following a 15° excitation pulse, a monopolar double-echo readout was acquired using flyback gradient pulses with 2 ms echo spacing. Only two echoes were acquired per radiofrequency excitation because EAT in mice at 9.4T can have $T_{2}^{*}$ values in the range of 5 ms, thus the signal-to-noise ratio (SNR) can be very low for additional echoes. Monopolar readouts were used to reduce the effects of eddy currents and gradient delays. Excitation and readout were repeated after each subsequent ECG trigger for approximately three times the maximum T_1_ of interest followed by RR interval pauses for two times the maximum T_1_ of interest to allow for complete magnetization recovery. Given a maximum T_1_ of interest of 1000 ms and an average mouse RR interval of 100 ms, 30 TI acquisitions with TI_1_ = 3.7 ms and ΔTI = RR interval were acquired followed by 20 RR pauses. This acquisition scheme was repeated for each radial spoke and again for each interleave. With each subsequent interleave, a delay of 0.2 ms was inserted prior to the double-echo readout, enabling the acquisition of various echo times. Using 10 interleaves, 20 TE images were acquired with effective echo spacing of 0.2 ms. The choice of TEs and echo spacing was chosen to minimize the variance in the estimates of the water and fat components as shown by Berglund et al. [28].

To create spatiotemporal incoherence of artifacts in the undersampled images, a golden-angle radial (stack-of-stars in the TE and TI dimensions) sampling scheme with golden-angle rotation applied to the in-plane spokes and across both TE and TI dimensions was used (Fig. 2B). For $N_{S}$ radial spokes, the projection angle for the i^th^ projection, n^th^ echo time, and m^th^ inversion time was calculated as:

(19) $$\begin{array}{c} \theta \left(i,n,m\right)=\mathrm{mod}\left(\left(i-1\right)\times \pi \times \frac{\sqrt{5}-1}{2},\pi \right)+\mathrm{mod}\left(\left(n-1\right)\times \frac{\pi }{N_{S}}\times \frac{\sqrt{5}-1}{2},\;\frac{\pi }{N_{S}}\right)\\ \;+\mathrm{mod}\left(\left(m-1\right)\times \frac{\pi }{N_{S}}\times \frac{\sqrt{5}-1}{2},\;\frac{\pi }{N_{S}}\right).\; \end{array}$$

### 3.2 Image reconstruction and parameter mapping

Image reconstruction and parameter mapping were implemented in MATLAB R2023a (MathWorks, Natick, Massachusetts). First, k-space trajectories for each radial spoke at each TE and TI were corrected for gradient delays along all spatial axes using independently acquired calibration data [29], [30]. Delays ranged from 2.1 to 2.6 µs, 3.2 to 3.4 µs, and −0.6 to 0.4 µs for the sagittal, coronal, and axial axes, respectively. To enable application of the method to multi-coil arrays, coil sensitivity maps and combination were performed using ESPIRiT with maps computed from the first TE and TI coil images [25]. Image reconstruction was performed using the HD-PROST-based framework as detailed in Section 2.4. A patch size of 3×3 and a patch stride of 1 were used. The reconstruction was performed over 5 ADMM iterations. The regularization parameter μ=0.75 was selected empirically and held constant across datasets.

Multi-TI images at the first TE were used to approximate T_1._ Due to heart rate (HR) variability during the scan, the different radial spokes of each TI image were acquired at slightly different TIs, resulting in clusters of TIs for each image. To account for this variation, as previously described, a fuzzy C-means clustering algorithm was used to determine the cluster of specific radial spokes corresponding to each TI, and the cluster centers were computed and used as the effective TIs [31]. Then, $T_{1}^{*}$, $A_{0}$, and $B_{\mathrm{diff}}$ were computed, followed by the determination of T_1_ using Eq. 2.

A conventional whole-image optimization algorithm was employed to estimate the spatially smooth field map, ψ, by minimizing Eq. 14 [32]. The transverse relaxation rate $R_{2}^{*}$ was determined through a brute-force search over discretized values from 0 to 500 s^−1^ in 0.5 s^−1^ increments. The initial phase, ϕ, was then calculated using Eq. 15. The parameter vector, x, containing water and fat components, was derived as previously described [16]. Once x was determined, ndb, nmidb, UFA, and PUFA ((4), (5), (6), (7)) were computed and used to calculate SFA and MUFA. The proton density fat fraction (PDFF), representing the proportion of total signal from fat protons, was calculated as F∕(F+W).

### 3.3 Phantom validation and selection of acceleration rate

Two phantoms were used to validate FAC and T_1_ mapping. The FAC phantom consisted of five 1 mL vials containing olive, sesame, and flaxseed oils, along with a 50/50 coconut/avocado oil mixture and a 25/75 coconut/sesame oil mixture, placed in a 5 mL conical tube of water. These oils were selected to span a broad FAC range comparable to in vivo adipose tissue [33]. The T_1_ phantom contained 1 mL vials of water with gadolinium (Gd) concentrations ranging from 0.1 to 0.5 mM for T_1_ mapping validation.

Joint FAC and T_1_ imaging was performed at 9.4T (Biospec 94/20, Bruker Biospin, Ettlingen, Germany) using a 600-bpm simulated ECG signal (SA Instruments, Inc., Stony Brook, New York,). Approximately fully sampled images (202 spokes) were acquired with the following parameters: slice thickness = 1 mm, field-of-view = 35 × 35 mm^2^, flip angle = 15°, acquisition matrix = 128 × 128, resolution = 0.27 × 0.27 mm^2^, bandwidth = 100 kHz, 30 TIs (TI_1_ = 3.7 ms, ΔTI = 100 ms), and 20 TEs (TE_1_ = 1.3 ms, ΔTE = 0.2 ms). Images were retrospectively undersampled such that the number of spokes used were Fibonacci numbers (from 144 to 1), guaranteeing uniform coverage of k-space [34]. A region of interest was drawn for each oil sample and the mean SFA fraction, MUFA fraction, PUFA fraction, PDFF, T_1,_ and $R_{2}^{*}$ were calculated. The PUFA fraction calculation assumes that fatty acids have at most two double bonds, which is valid for approximately 98% of adipose tissue fatty acids but not for certain plant oils containing significant triunsaturated fatty acids. To account for this, the PUFA fraction was adjusted using a fixed triunsaturated fatty acid fraction ($F_{\mathrm{TRIFA}}$) measured by NMR for each oil, by applying the formula $\mathrm{PUFA}=\frac{\mathrm{nmidb}}{3}-F_{\mathrm{TRIFA}}$. Mean absolute error (MAE) was computed between fully sampled and retrospectively undersampled SFA fraction, MUFA fraction, PUFA fraction, PDFF, T_1_, and $R_{2}^{*}$ maps for all acceleration rates. The structural similarity (SSIM) index [35] was computed between denoised fully sampled and denoised retrospectively undersampled images over the entire oil phantom region, excluding background pixels.

Reference SFA fraction, MUFA fraction, and PUFA fraction were determined by nuclear magnetic resonance (NMR) spectroscopy as previously described [16]. Reference T_1_ values were determined using a conventional ECG-gated inversion recovery T_1_ mapping method with the following acquisition parameters: slice thickness = 1 mm, field-of-view = 35 × 35 mm^2^, flip angle = 1°, acquisition matrix = 128 × 128, resolution = 0.27 × 0.27 mm^2^, bandwidth = 100 kHz, 30 TIs (TI_1_ = 3.7 ms, ΔTI = 100 ms). Agreement between joint FAC and T_1_ values and reference methods across acceleration rates was evaluated using linear regression, with Pearson’s correlation coefficients (*r*) and *p*-values assessing linearity and significance.

### 3.4 In vivo MRI protocol and image analysis

All animal studies were performed in accordance with protocols that conformed to the Declaration of Helsinki as well as the Guide for Care and Use of Laboratory Animals [36] and were approved by the Animal Care and Use Committee at the University of Virginia. Mice were maintained at the University of Virginia Center for Comparative Medicine pathogen-free vivarium facility. CMR was performed on a 9.4T system using a ^1^H transmit-receive quadrature volume radiofrequency coil (35 mm inner diameter, Bruker BioSpin GmbH, Ettlingen, Germany). During imaging, mice were anesthetized with 1.25% isoflurane, and core temperature was maintained at 36 ± 0.5 °C using circulating warm air. The ECG, body temperature, and respiration were monitored (SA Instruments, Inc., Stony Brook, New York). Localizer imaging was performed to establish a mid-ventricular axial slice with sufficient EAT. Undersampled inversion recovery multi-echo images for joint FAC and T_1_ mapping were acquired using the pulse sequence described in Section 3.1. Acquisition parameters included: slice thickness = 1 mm, field-of-view = 25 × 25 mm^2^, flip angle = 15°, acquisition matrix = 128 × 128, resolution = 0.2 × 0.2 mm^2^, bandwidth = 100 kHz, $N_{S}$ = 21, 30 TIs (TI_1_  = 3.7 ms, ΔTI = RR interval ms), and 20 TEs (TE_1_ = 1.3 ms, ΔTE = 0.2 ms), with a total scan time of approximately 17 min.

Images and parameter maps were reconstructed as described in Section 3.2. The EAT and SAT were manually segmented using the images of total fat content F. To reduce partial volume effects and ensure that regions of interest were predominantly adipose tissue, we excluded border pixels and pixels with PDFF < 50%. Additionally, mice with < 30 pixels in the region of interest were excluded from analysis to prevent bias from small depots with potentially significant partial volume effects. The mean parameter values were calculated for each depot, and EAT parameter indexes were computed as the ratio of the mean EAT to SAT values for SFA fraction, MUFA fraction, PUFA fraction, and PDFF. These EAT quality metrics were indexed to their SAT counterparts as SAT is considered a metabolically healthier adipose depot [37], [38], [39], [40]. Thus, the ratio utilized an intra-subject reference and accounted for inter-subject differences in overall inherent adipose physiology (e.g., lipid metabolism, deposition, and synthesis) [33], [38]. Relaxation parameters, T_1_ and $R_{2}^{*}$, were not indexed, as they reflect more than differences in physiology, such as local field inhomogeneities.

### 3.5 Application of joint FAC and T_1_ mapping to mouse models of metabolic heart disease with differing EAT FAC and inflammatory profiles

This study applied joint FAC and T_1_ mapping to investigate relationships between MRI-derived EAT parameters and tissue markers of inflammation, including adipocyte size, macrophage infiltration, and proinflammatory cytokines. To generate a range of adipose tissue compositions, three groups of C57Bl/6J mice (n = 16–20 mice/group (Jackson Laboratories, Bar Harbor, Maine); strain #000664) were studied: (1) mice fed a high-fat high-sucrose diet (HFHSD) (40% kcal fat, 40% kcal sucrose; Diet 123727, Research Diets, Inc., New Brunswick, New Jersey,), (2) mice fed an HFHSD plus the SGLT2i, empagliflozin (EMPA) (40% kcal fat, 40% kcal sucrose, 30 mg/kg/day EMPA; Diet 21011406, Research Diets, Inc), and (3) mice fed a high-fat diet (HFD) (60% kcal fat; Diet 12492, Research Diets, Inc.). Diets began at 6–8 weeks of age and continued for 18 weeks.

These groups were selected to induce varying metabolic conditions, visceral adipose tissue inflammation, EAT development, and distinct FAC profiles across various adipose depots. The HFD consisted of 245 g lard (40% SFA, 45% MUFA, 10% PUFA) and 25 g soybean oil (15% SFA, 23% MUFA, 58% PUFA), resulting in an overall composition of 39% SFA, 45% MUFA, and 16% PUFA. The HFHSD included 135 g coconut oil (94% SFA, 5% MUFA, 1% PUFA) and 45 g soybean oil (15% SFA, 23% MUFA, 58% PUFA), yielding 75% SFA, 10% MUFA, and 15% PUFA. HFD-fed mice were expected to exhibit a lower SFA fraction and a higher MUFA fraction, while HFHSD-fed mice were expected to show a higher SFA fraction and a lower MUFA fraction. Though designed to modulate FAC composition, both HFHSD and HFD mice develop visceral adipose tissue inflammation and metabolic heart disease [41]. EMPA was included due to its known effects on visceral adipose tissue, including reducing inflammation, adipose tissue browning, and altering lipid metabolism via decreased lipogenesis and increased lipolysis [15], [42]. The addition of EMPA to HFHSD-fed mice allowed for sufficient weight gain and EAT development while modifying EAT FAC and reducing inflammation.

### 3.6 Histology

EAT was collected after CO_2_-induced death and fixed in 4% PFA in PBS for 7–10 days at 4 °C. EAT was paraffin embedded and cryosectioned at 5 µm thickness and mounted. Slides were deparaffinized. Briefly, sections were submerged in xylene (3 min), 1:1 xylene:ethanol (3 min) 100% ethanol (2 × 3 min), 95% ethanol (3 min), 70% ethanol (3 min), and 50% ethanol (3 min). Antigen retrieval was performed using citrate-based solution (Vector Laboratories H-330, Newark, California) where slides were submerged in antigen retrieval solution and heated to boiling for 20 min, slides were then cooled for 1 h at room temperature. Tissue sections were then blocked for 1 h in antibody blocking buffer (FGS, donkey serum) at room temperature. Antibody blocking buffer was removed and replaced with antibody blocking buffer containing primary antibody overnight at 4 °C. Sections were then washed (PBS+FGS+Tween for 5 min, 2× PBS 5 min) and incubated in antibody blocking buffer containing secondary antibody (1:100) for 1 h at room temperature protected from light. Sections were washed (3xPBS) and counterstained with DAPI (Thermo Fisher Scientific D3571, Waltham, Massachu) befre mounting. Sections were imaged on an Olympus Fluoview 1000 and are representative images of composite z-stacks. Analysis (thresholding and manual counting) was performed in ImageJ.

### 3.7 Cytokine assays

Hearts and EAT were snap frozen in liquid nitrogen prior to sample preparation after CO_2_-induced death. Hearts and EAT were homogenized in 1:1 PBS and Cell Lysis buffer (R&D Systems 895347, Minneapolis, Minnesota) by bead homogenization. Around 200 μL of tissue lysate was provided for Luminex analysis with the Flow Cytometry Core at the University of Virginia School of Medicine. A standard mouse 32-plex Luminex cytokine panel was used to assess both proinflammatory and anti-inflammatory signaling (Supplemental Table 1). Cytokines that were not detected in the majority of samples were excluded from analysis. Each sample was run in triplicate and quantification was performed relative to a standard curve for each independent cytokine. Each cytokine was normalized to total protein per individual sample.

### 3.8 Statistics

All statistical analyses were performed using GraphPad Prism 10.4.0 (GraphPad Software, La Jolla, California). Group comparisons for EAT and SAT parameters were conducted using one-way analysis of variance with Fisher’s least significant difference post hoc tests to assess differences among groups. Spearman correlation coefficients (*r*) and associated p-values were calculated to evaluate associations between MRI-derived parameters and tissue markers of inflammation. A significance threshold of p<0.05 was used.

## 4 Results

### 4.1 Phantom validation and selection of acceleration rate

**Fig. 3:**
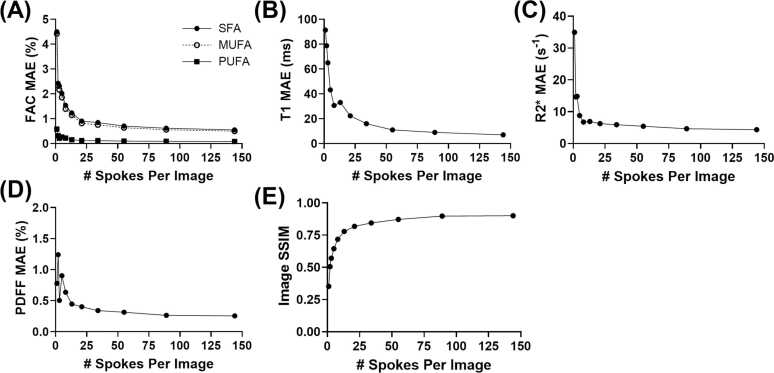
Error analysis of retrospectively undersampled images. MAE between (A) SFA, MUFA, and PUFA fractions, (B) T_1_, (C) $R_{2}^{*}$, and (D) PDFF values computed from retrospectively undersampled phantom images and fully sampled acquisitions. (E) SSIM index between denoised retrospectively undersampled and fully sampled images. *FAC* fatty acid composition, *PDFF* proton density fat fraction, *SFA* saturated fatty acid, *MUFA* monounsaturated fatty acid, *PUFA* polyunsaturated fatty acid, *MAE* mean absolute error, *SSIM* structural similarity

To determine the optimal acceleration rate, an error analysis was performed comparing accelerated acquisitions to fully sampled images and maps. Rate-9.6 acceleration (21 spokes per image) was identified as optimal, achieving a scan time of approximately 17 min while maintaining low MAEs of 0.91% for SFA fraction, 0.82% for MUFA fraction, 0.12% for PUFA fraction, 0.40% for PDFF, 22.36 ms for T_1_, and 6.26 s^−1^ for $R_{2}^{*}$ with an SSIM > 0.80 (Fig. 3).

**Fig. 4:**
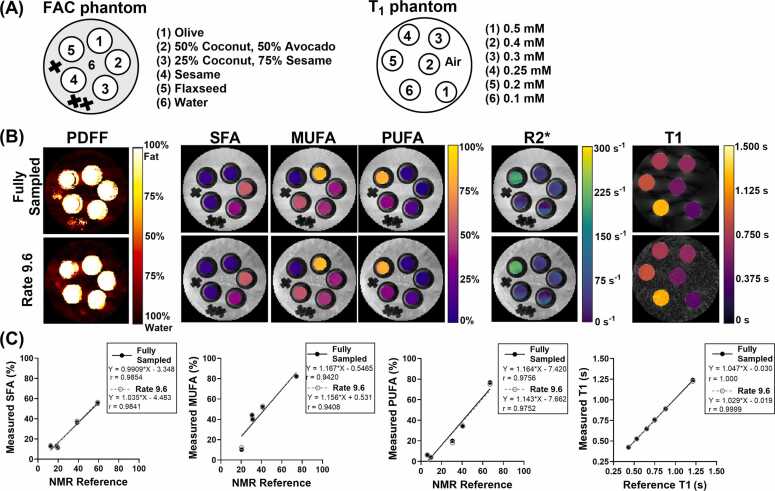
Phantom layout and parameter maps. (A) Layout of oil and gadolinium phantoms used for FAC and T_1_ validation, respectively. The FAC phantom consists of five 1 mL oil mixtures submerged in 5 mL of water, while the T₁ phantom contains six 1 mL vials of water with Gd concentrations ranging from 0.1 to 0.5 mM. (B) Parameter maps of PDFF, SFA/MUFA/PUFA fractions, $R_{2}^{*}$, and T_1_ for fully sampled and rate-9.6 accelerated acquisitions, overlaid on the phantom images. (C) Linear regression analysis between joint FAC and T_1_ measured values and reference values for fully sampled and rate-9.6 accelerated acquisitions. Pearson *r* values are reported. *Gd* gadolinium, *FAC* fatty acid composition, *PDFF* proton density fat fraction, *SFA* saturated fatty acid, *MUFA* monounsaturated fatty acid, *PUFA* polyunsaturated fatty acid

Next, the accuracy of joint FAC and T_1_ estimations was validated against NMR (FAC) and conventional MRI T_1_ mapping. Fig. 4B shows fully sampled and rate-9.6 undersampled parameter maps in the FAC and T_1_ phantoms. Phantom validation demonstrated strong correlations (*r*>0.94, p<0.05) between reference and measured values, confirming the accuracy of the joint method (Fig. 4C). For fully sampled acquisitions, regression analysis yielded slopes of 0.99, 1.17, and 1.16 with biases of −3.35, −0.55, and −7.42 for SFA, MUFA, and PUFA fractions respectively, and a slope of 1.05 with a bias of 0.03 for T_1._ For rate-9.6 accelerated acquisitions, regression slopes remained similar at 1.04, 1.16, and 1.14 with biases of −4.5, −0.53, and −7.66 for SFA, MUFA, and PUFA fractions, and a slope of 1.03 with a bias of −0.02 for T_1_.

MAEs between fully sampled joint FAC and T_1_ mapping values and reference measurements were 4.02% (SFA), 10.15% (MUFA), and 6.28% (PUFA) compared to NMR, and 8.66 ms for T_1_ compared to reference T_1_ mapping. For rate-9.6 accelerated acquisitions, MAEs remained similar at 3.57% (SFA), 10.03% (MUFA), 6.49% (PUFA), and 7.71 ms for T_1_, demonstrating that acceleration maintains measurement accuracy.

### 4.2 Accelerated in-vivo images and parametric mapping

**Fig. 5:**
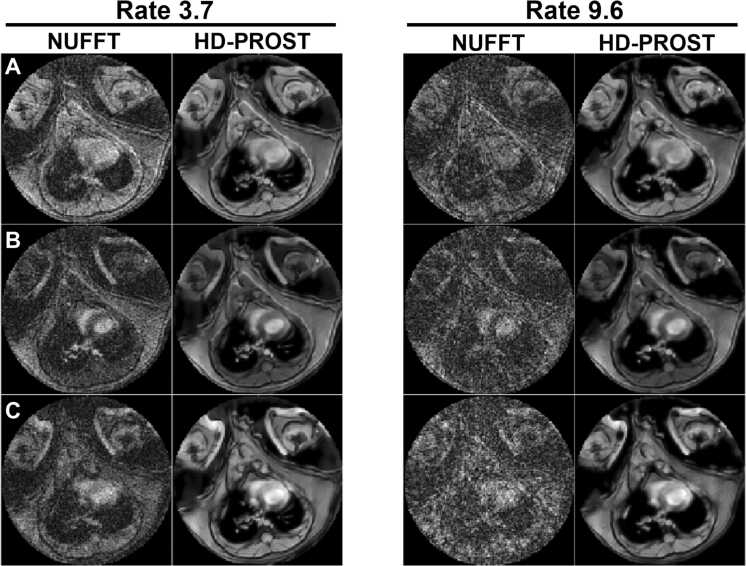
Comparison of undersampled and HD-PROST reconstructed in vivo images. Nonuniform fast Fourier transform undersampled and HD-PROST reconstructed images acquired at acceleration rates 3.7 and 9.6 for (A) TE_1_, TI_1_, (B) TE_1_, TI_15_, and (C) TE_4_, TI_30_ in the same mouse with high-fat high-sucrose diet-induced obesity. *HD-PROST* high-dimensionality undersampled patch-based reconstruction, *TI* inversion times, *TE* echo times

Example NUFFT- and HD-PROST-reconstructed images at multiple TEs and TIs are shown in Fig. 5 for two different acceleration rates. While phantom imaging allowed for fully sampled reference images, the associated scan time (∼2.8 h) was not feasible for in vivo mouse imaging. Instead, a 55-spoke acquisition (46 min, rate-3.7) followed by a 21-spoke acquisition (17 min, rate-9.6) was performed in the same mouse to compare image quality. This combined scan time represented the longest feasible in vivo imaging duration. The 21-spoke acquisition was chosen based on phantom analysis determining the optimal acceleration rate. Although the undersampled images exhibit substantial noise-like artifacts, the reconstruction effectively reduces these artifacts while preserving image quality. The rate-9.6 acquisition produces images comparable to the highly sampled rate-3.7 acquisition, with an SSIM of 0.91 computed over the mouse body, supporting the choice of rate-9.6 for in vivo imaging.

**Fig. 6:**
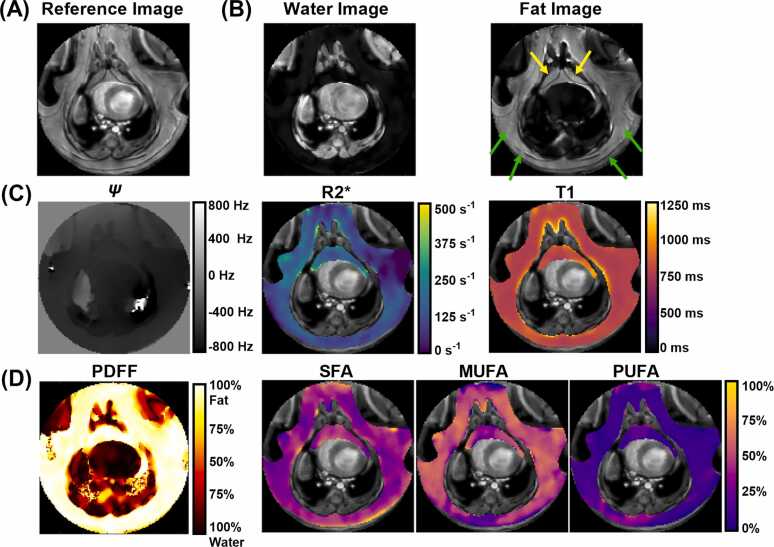
In vivo parametric maps from a representative HFHSD mouse. Images from a mid-ventricular axial slice showing: (A) T_1_-weighted reference image, (B) water (*W*) and fat (*F*) signal images (yellow arrows = EAT, green arrows = SAT), (C) maps of off-resonance (ψ), $R_{2}^{*}$, and T_1_, and (D) fat parameter maps including PDFF, SFA fraction, MUFA fraction, and PUFA fraction. Parameter maps are overlaid on manually contoured EAT and SAT regions. *HFHSD*, high-fat high-sucrose diet. *EAT* epicardial adipose tissue, *SAT* subcutaneous adipose tissue, *PDFF* proton density fat fraction, *SFA* saturated fatty acid, *MUFA* monounsaturated fatty acid, *PUFA* polyunsaturated fatty acid

Example rate-9.6 accelerated in vivo parameter maps are shown in Fig. 6. Water (W) and fat (F) signal images, and maps of PDFF and off-resonance (ψ) are displayed, along with $R_{2}^{*}$, T_1_, SFA fraction, MUFA fraction, and PUFA fraction maps overlaid on EAT and SAT regions.

### 4.3 Joint FAC and T_1_ mapping detects diet and SGLT2-inhibitor-induced differences in EAT and SAT properties

**Fig. 7:**
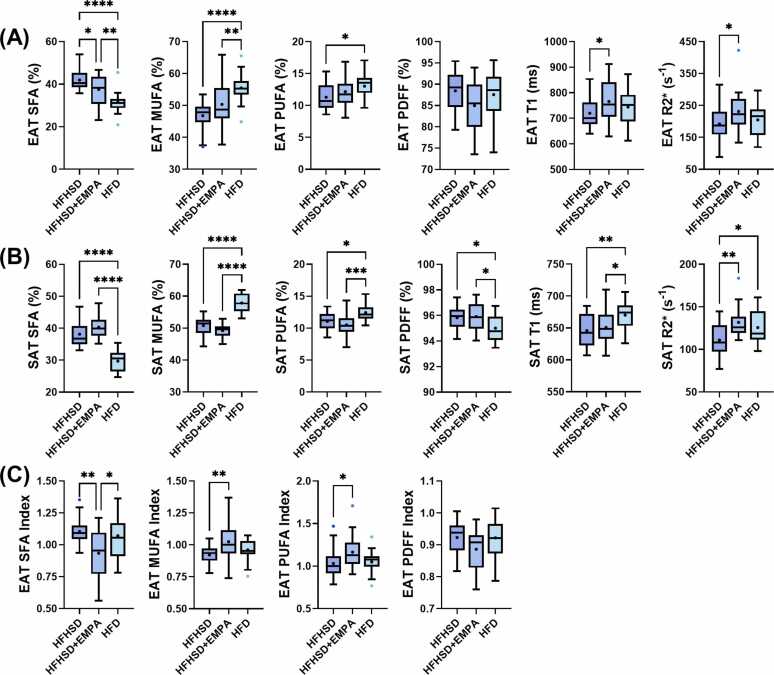
Diet and SGLT2 inhibitor-induced differences in adipose tissue MRI parameters. Tukey boxplots of absolute SFA fraction, MUFA fraction, PUFA fraction, PDFF, T_1_, and $R_{2}^{*}$ values for (A) EAT and (B) SAT in mice fed an HFHSD (n = 20), HFHSD+EMPA (n = 17), or HFD (n = 16) for 18 weeks. (C) EAT index values for SFA, MUFA, PUFA, and PDFF in the same groups. *p<0.05, **p<0.01, ***p<0.001, and ****p<0.0001. *HFHSD*, high-fat high-sucrose diet; *EMPA*, empagliflozin; *HFD*, high-fat diet. *EAT* epicardial adipose tissue, *SAT* subcutaneous adipose tissue, *PDFF* proton density fat fraction, *SFA* saturated fatty acid, *MUFA* monounsaturated fatty acid, *PUFA* polyunsaturated fatty acid, *SGLT2* sodium-glucose cotransporter-2 inhibitor

Absolute EAT SFA, MUFA, PUFA, PDFF, T_1_, and $R_{2}^{*}$ results for each group of mice are summarized in Fig. 7A. The average EAT region of interest sizes were 114.2 ± 75.2, 157.6 ± 68.1, and 222.0 ± 116.0 pixels for the HFHSD+EMPA, HFD, and HFHSD groups, respectively, corresponding to cross-sectional areas of approximately 4.6 ± 3.0 mm^2^, 6.3 ± 2.7 mm^2^, and 8.9 ± 4.6 mm^2^. EAT FAC profiles differed significantly across all groups. HFHSD mice had the highest EAT SFA faction and lowest EAT MUFA fraction, with significantly higher SFA fraction compared to HFHSD+EMPA (p<0.05) and HFD (p<0.0001) mice, and significantly lower MUFA (p<0.0001) and PUFA (p<0.05) fraction compared to HFD mice. Additionally, HFHSD+EMPA mice exhibited higher EAT SFA fraction (p<0.01) and lower MUFA fraction (p<0.01) than HFD mice. HFHSD+EMPA mice exhibited significantly higher $R_{2}^{*}$ (p<0.05) and T_1_ (p<0.05) in the EAT compared to HFHSD mice.

SAT parameter results are summarized in Fig. 7B. HFD mice had the lowest SAT SFA fraction and highest SAT MUFA fraction with significant differences compared to HFHSD (p<0.0001) and HFHSD+EMPA (p<0.0001) mice. HFD mice also exhibited an elevated SAT PUFA fraction compared to HFHSD+EMPA (p<0.001) and HFHSD (p<0.05) mice. SAT PDFF was the lowest in the HFD mice compared to both HFHSD (p<0.05) and HFHSD+EMPA mice (p<0.05). HFD mice also exhibited the longest SAT T_1_ compared to HFHSD (p<0.01) and HFHSD+EMPA (p<0.05) mice. HFHSD mice had lower SAT $R_{2}^{*}$ compared to HFHSD+EMPA (p<0.01) and HFD (p<0.05) mice.

The EAT parameters indexed to those of SAT were compared between groups. The indexed parameters revealed a distinct profile, with HFHSD+EMPA mice displaying significantly reduced EAT SFA index compared to HFHSD (p<0.01) and HFD (p<0.05) mice (Fig. 7C). HFHSD+EMPA mice also exhibited a significantly higher MUFA (p<0.01) and PUFA (p<0.05) index than HFHSD mice.

### 4.4 MRI biomarkers of EAT correlate with histological and cytokine markers of inflammation

**Fig. 8:**
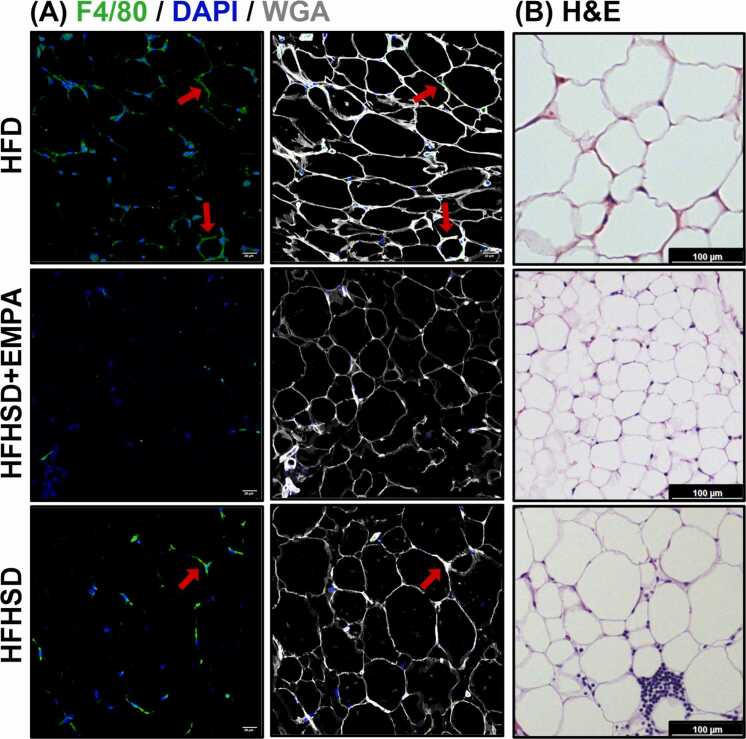
Epicardial adipose tissue histology. (A) F4/80 (green) and DAPI (blue) staining show increased macrophage infiltration in HFD and HFHSD mice, which is reduced with EMPA treatment (left column). Merged F4/80, DAPI, and WGA (white) images highlight crown-like structures of macrophages surrounding adipocytes (red arrows) in HFD and HFHSD conditions (middle column). Scale bar: 20 µm. (B) Hematoxylin and eosin (H&E) staining reveals larger adipocytes in HFD and HFHSD conditions, while EMPA-treatment displays smaller, more uniform adipocytes. Images in (A) and (B) were obtained from different mice within the same experimental groups. Scale bar: 100 µm. *WGA*, wheat germ agglutinin. *HFHSD* high-fat high-sucrose diet, *EMPA* empagliflozin, *HFD* high-fat diet

To investigate associations between EAT MRI parameters and tissue-level markers of inflammation, we analyzed correlations between MRI-derived EAT indexes and histological and cytokine measures in both EAT and cardiac tissue. Fig. 8 presents representative EAT images depicting varying inflammatory phenotypes from each mouse group stained for F4/80+ macrophages and hematoxylin and eosin for adipocyte morphology. Macrophage infiltration is higher in the HFD and HFHSD groups but reduced with EMPA. Hematoxylin and eosin staining reveals larger adipocytes in HFD and HFHSD mice, while EMPA-treated mice show smaller, more uniform adipocytes.

**Fig. 9:**
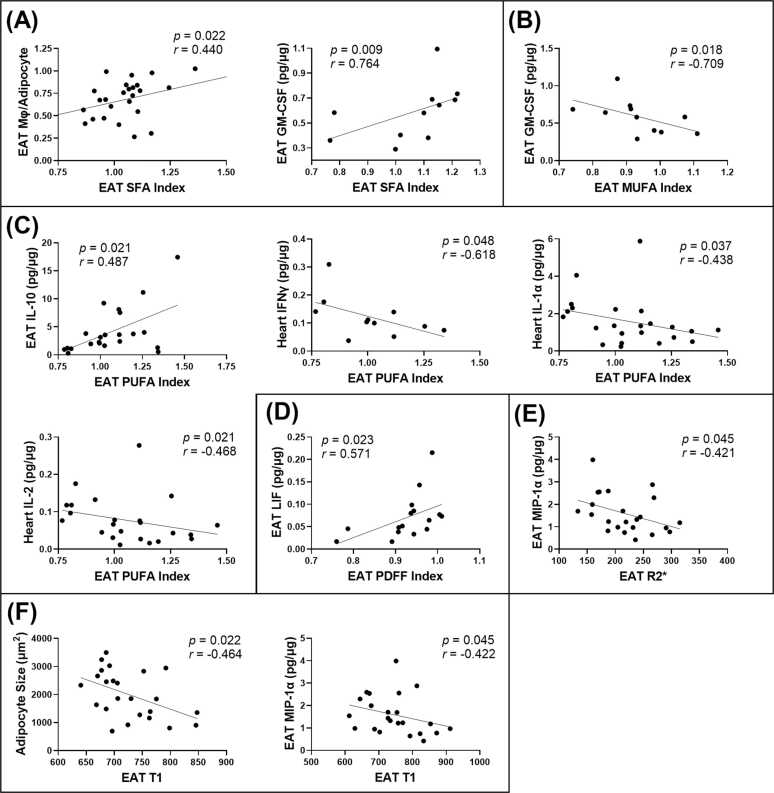
Relationships between EAT MRI parameters and tissue inflammation markers. Correlations between EAT MRI parameters – (A) SFA index, (B) MUFA index, (C) PUFA index, (D) PDFF index, (E) $R_{2}^{*}$, and (F) T_1_ – and tissue measures, including macrophage density (Mφ/adipocyte), adipocyte size (µm²), and cytokine levels (pg/µg total protein). Spea*r*man’s *r* and *p*-values are reported for each plot. *HFHSD*, high-fat high-sucrose diet. *EAT* epicardial adipose tissue, *SAT* subcutaneous adipose tissue, *FAC* fatty acid composition, *PDFF* proton density fat fraction, *SFA* saturated fatty acid, *MUFA* monounsaturated fatty acid, *PUFA* polyunsaturated fatty acid

Significant (p<0.05) relationships between in vivo MRI parameters (SFA, MUFA, PUFA, PDFF indexes, $R_{2}^{*}$, and T_1_) and ex vivo tissue measurements are shown in Fig. 9. EAT SFA index positively correlated with EAT macrophage infiltration, assessed as the ratio of macrophages per adipocyte (*r *= 0.440, p = 0.022), and granulocyte-macrophage colony-stimulating factor (GM-CSF) levels in the EAT (*r *= 0.764, p = 0.009). In contrast, EAT MUFA index negatively correlated with GM-CSF levels in the EAT (*r* = −0.709, p = 0.018). EAT PUFA index was positively correlated with interleukin (IL)−10 (*r* = 0.487, p = 0.021) in the EAT, and negatively correlated with cytokine expression in the heart, including interferon gamma (IFN-γ) (*r* = −0.618, *p *= 0.048), IL-1α (*r* = −0.438, *p *= 0.037), and IL-2 (*r* = −0.468, p = 0.021). EAT PDFF index showed a positive correlation with leukemia inhibitor factor (LIF) (*r* = 0.571, p = 0.023) levels in the EAT. Among MRI relaxation parameters, EAT T_1_ negatively correlated with EAT adipocyte size (*r* = −0.464, p = 0.022) and macrophage inflammatory protein 1-alpha (MIP-1α) levels in the EAT (*r* = −0.422, p = 0.045). EAT $R_{2}^{*}$ also negatively correlated EAT MIP-1α levels (*r* = −0.421, p = 0.045). No other significant associations with inflammatory markers were observed.

## 5 Discussion

This study developed an accelerated method for joint FAC and relaxation parameter mapping of EAT, expanding upon a growing body of research aimed at noninvasively characterizing EAT. By integrating relaxation parameter mapping and FAC, this method provides a more comprehensive assessment of EAT quality, as both adipose tissue FAC and relaxation parameters are altered in chronic metabolic inflammatory states, such as obesity. The approach exploits correlations across multiple contrast dimensions while leveraging golden-angle radial sampling and an HD-PROST-based reconstruction framework to achieve high acceleration rates. To our knowledge, this is the first study to correlate in vivo CMR-derived EAT parameters with ex vivo markers of inflammation, providing evidence that these MRI parameters represent noninvasive biomarkers of proinflammatory EAT.

Other imaging modalities, such as CT and PET have also been investigated for characterizing EAT. 18F-FDG PET detects metabolic activity and has been used as a surrogate marker of EAT inflammation, with reported associations in coronary artery disease and atrial fibrillation [6], [43], [44]. CT-based methods, including coronary CT angiography, can quantify EAT density using the fat attenuation index, which has been shown to correlate with PET-assessed inflammation [45]. In contrast to these approaches, our CMR-based method quantifies FAC, including SFAs, which are central to the biology of adipose tissue inflammation. As such, CMR-derived indices may offer a more specific and biologically interpretable assessment of proinflammatory EAT.

This method builds upon prior CMR parameter mapping of adipose tissue and is situated within a broader context of multiparametric CMR methods that leverage low-rank structure to accelerate imaging and improve mapping quality [46], [47], [48], [49]. While several groups have demonstrated in vivo adipose tissue FAC mapping alone [16], [17], [21], [50], [51], joint mapping of FAC and T_1_ has yet to be developed for cardiac applications. Preclinical EAT FAC mapping at 7T demonstrated feasibility in mouse models of coronary microvascular disease [17], yet this approach relied on Cartesian sampling, limiting its robustness to motion and requiring longer scan times. The proposed method improves upon these limitations by employing golden-angle radial sampling, which provides increased robustness to motion artifacts and greater incoherence of undersampling artifacts. We employed an HD-PROST reconstruction to leverage local low-rank properties across spatial and contrast dimensions to suppress incoherent undersampling artifacts, support high acceleration rates, and achieve high SNR. Others also recently utilized the coil dimension along with spatial and contrast dimensions to further exploit high-dimensional low-rank structure, which is an innovative method that could be employed in the future to help translate the present method for clinical imaging [49]. Joint parameter mapping has been explored for simultaneous quantification of tissue relaxation parameters and fat/water separation in the heart [52], [53], [54], though primarily for myocardial T_1_ mapping and EAT volume rather than EAT quality characterization [54], [52]. Ostenson et al. recently demonstrated the feasibility of joint triglyceride saturation and water T_1_ mapping in periclavicular adipose depots at 3T [55], but directly extending this to preclinical EAT imaging presents challenges due to cardiac and respiratory motion, high heart rates (approximately 600-bpm), and the relatively small size of the EAT depot. Our work addresses these challenges by developing an ECG-gated inversion recovery multi-echo sequence with golden-angle rotation through multiple contrast dimensions with a higher-order low-rank reconstruction and fuzzy C-means clustering of TIs.

Phantom validation studies were conducted to assess the accuracy of the method for quantifying FAC and T_1_. Due to the long scan times required for fully sampled in vivo acquisitions, direct comparisons between fully sampled and accelerated images were not feasible. Instead, validation was performed in phantoms with simulated ECG signals, allowing fully sampled acquisitions for accurate assessment. Phantom measurements demonstrated strong correlations with reference values obtained from NMR spectroscopy for FAC and standard inversion recovery CMR for T_1_.

This study identified distinct diet- and EMPA-induced differences in CMR-derived adipose tissue parameters. Given that subcutaneous and visceral adipose tissue triglyceride fatty acid profiles reflect dietary fat composition [10], [56], [57], [58], [59], the 18-week duration of dietary exposure likely allowed substantial incorporation of dietary fatty acids into both of these expanding adipose depots. As a result, our method detected expected differences in absolute FAC parameters between mice fed an HFD (high in MUFA) and an HFHSD (high in SFA), validating expected in vivo shifts in adipose tissue composition. While the observed changes were modest, they are consistent with prior studies reporting FAC shifts of 5–7% with dietary interventions in mice and large animals [10], [60]. Indexing to SAT, which accounts for the propensity to store, mobilize, and endogenously synthesize lipid in different depots under different conditions [33], [38], revealed a distinct EMPA-treated phenotype. HFHSD+EMPA mice exhibited a low EAT SFA index and high EAT MUFA and PUFA indexes, consistent with known anti-inflammatory effects of SGLT2i on adipose tissue. In contrast, HFHSD and HFD mice, both prone to developing proinflammatory adipose tissue and metabolic heart disease, had similar indexed values, with high EAT SFA indexes and low EAT MUFA and PUFA indexes. These findings suggest that MRI-derived parameters may serve as biomarkers for adipose tissue properties that are modifiable with dietary or pharmacologic interventions. While SGLT2i treatment also led to modest changes in EAT FAC, a prior study evaluating eplerenone led to greater changes in FAC [17], demonstrating the ability of MRI FAC to detect both large and moderate changes due to pharmacological interventions.

Using ex vivo measurements of various tissue parameters, our study provides evidence that MRI-derived FAC indices and relaxation parameters are noninvasive biomarkers of proinflammatory EAT in metabolic heart disease. The EAT SFA index positively correlated with macrophage infiltration and GM-CSF, a proinflammatory cytokine that is known to promote macrophage recruitment and has been implicated in adipose inflammation and heart failure [61], [62]. Macrophages are key mediators of adipose tissue inflammation, sustaining chronic inflammation in obesity through alternative polarization, crown-like structure formation, cytokine secretion, and immune cell recruitment [63]. The PDFF index was positively associated with LIF, which, although protective during acute stress, is linked to worsening cardiac function when chronically elevated [64], [65], [66]. In contrast, higher relative fatty acid unsaturation in the EAT was associated with anti-inflammatory properties. The MUFA index negatively correlated with GM-CSF, while the PUFA index positively correlated with the potent anti-inflammatory cytokine IL-10 in the EAT [67], [68], and negatively correlated with major proinflammatory cytokines in the heart, including IFN-γ, IL-1α, and IL-2 [69], [70], [71].

Relaxation parameters, T_1_ and $R_{2}^{*}$, serve as additional noninvasive markers of EAT quality. EAT T_1_ was negatively correlated with adipocyte size and the proinflammatory cytokine MIP-1α (or CCL3), a macrophage-derived mediator of adipose tissue inflammation linked to both obesity and reduced left ventricular function in heart failure patients [72], [73]. While increased lipid content likely contributes to T_1_ shortening, other factors such as elevated oxidative stress, changes in fatty acid composition, lower temperature, and local hypoxia may also play a role [74]. EAT $R_{2}^{*}$ similarly inversely correlated with MIP-1α, suggesting that increased $R_{2}^{*}$ may reflect a healthier EAT phenotype. Notably, elevated $R_{2}^{*}$ has been identified as a biomarker of increased iron content in brown adipose tissue, a more metabolically active adipose subtype with beneficial effects on obesity and inflammation [75].

Taken together, these results indicate that increases in EAT SFA and PDFF indexes, coupled with reduced MUFA and PUFA indexes, T_1_, and $R_{2}^{*}$, characterize a proinflammatory EAT phenotype marked by adipocyte hypertrophy, macrophage infiltration, and local inflammatory signaling in the setting of cardiometabolic dysfunction. These relationships support the use of CMR-based EAT parametric mapping as a tool for identifying proinflammatory EAT phenotypes that may be modifiable through dietary or pharmacologic interventions.

Future work may focus on translating this method to human imaging and evaluating the clinical utility of these biomarkers. Although lower field strengths and a shorter total data sampling time for human imaging will reduce SNR, these reductions are easily offset by the approximately 800-fold increase in voxel size for clinical vs. mouse imaging, resulting in highly favorable SNR for human imaging compared to mouse imaging. Clinical implementation of the method will require adaptations to sequence timing—such as increased echo spacing, longer echo trains, and modified T_1_ sampling—but a free-breathing, motion-compensated technique is feasible given the longer $T_{2}^{*}$ and shorter T_1_ of EAT at lower clinical field strengths, and slower heart rate of humans. We estimate that a free-breathing method could have a scan time of approximately 2 min per slice. Clinical studies are needed to validate these EAT biomarkers in various cardiometabolic scenarios, to assess their relationships with cardiovascular functional parameters, and to investigate their diagnostic and prognostic potential. Successful translation of this work may support early identification of at-risk patients and enable more personalized therapeutic approaches in coronary artery disease, coronary microvascular disease, heart failure, atrial fibrillation, and other types of heart disease where EAT is known to play a detrimental role.

## 6 Limitations

Our study has several limitations. First, the imaging protocol was optimized for the measurement of T₁ values for adipose tissue, precluding accurate measurements in other tissues of potential interest, such as the myocardium. While not the focus of the study, future work could optimize methods for imaging of both the EAT and myocardium. Another potential limitation is the reliance on assumptions in the signal model. Using a fixed fatty acid chain length for all in vivo measurements may introduce errors if actual chain lengths deviate from this assumption. While accurate for HFHSD-fed mice, it may be less so for others. However, given the small expected physiological variation in chain length, a single model avoids potential bias from different assumptions for each group. Furthermore, while a Look-Locker correction was applied for T₁ estimation, it remains an approximation, and T₁ measurements may be affected by B₁ inhomogeneities, imperfect adiabatic inversion pulses, and deviations in signal relaxation due to repetitive readouts. The proposed method does not differentiate between the T₁ of water and fat. Although this was not a primary focus of the present study, future implementations could work toward enabling independent assessment of fat and water T₁ contributions to EAT inflammation, with potential to expand this model to include different T_1_ values for each fat resonance. Additionally, we used oil phantoms instead of EAT for CMR FAC validation by NMR spectroscopy. As the amount of EAT available from mice for ex vivo studies was limited, we prioritized using this tissue for histology and flow cytometry instead of NMR spectroscopy. Although MRI FAC measurements in phantoms strongly correlated with reference values, biases in SFA, MUFA, and PUFA measurements may limit sensitivity to subtle changes in FAC. These errors could be reduced in future work through improved B_0_ mapping, increasing the number of echo times, respiratory motion compensation, and increased SNR. While this study demonstrated promising correlations between CMR-derived metrics and tissue markers of inflammation, these correlations exhibited substantial scatter. This scatter was likely due to the small size of mouse EAT samples, leading to variability in cytokine measurements.

## 7 Conclusions

This study introduces an accelerated CMR approach for joint mapping of FAC and relaxation parameters in EAT, enabling noninvasive, multiparametric assessment of adipose tissue quality. By combining golden-angle radial sampling across multiple time dimensions with an iterative higher-order low-rank reconstruction, the approach substantially reduces scan time while preserving accuracy, as demonstrated in both phantom and in vivo validation. Comparisons between CMR-derived and tissue measurements support EAT T_1_ and $R_{2}^{*}$, and SFA, MUFA, PUFA, and PDFF indexes as noninvasive biomarkers of proinflammatory EAT in metabolic heart disease. These noninvasive biomarkers may serve as valuable tools in future research aimed at evaluating therapeutic interventions that target proinflammatory EAT.

## Funding

10.13039/100000050National Heart, Lung, and Blood Institute (R01 HL162872), Bethesda, Maryland; National Institutes of General Medical Sciences Medical Scientist Training Grant Program T32GM007267, Bethesda, Maryland; American Heart Association Pre-Doctoral Fellowship 23PRE1011280, Dallas, Texas; National Institutes of Health Instrumentation Grant Program S10OD025024, Bethesda, Maryland; National Heart, Lung, and Blood Institute Cardiovascular Science Training Grant Program (T32 HL007284), Bethesda, Maryland.

## Author contributions

**Julia E. Bresticker:** Writing – review & editing, writing – original draft, visualization, validation, software, methodology, investigation, formal analysis, conceptualization. **Caitlin M. Pavelec:** Writing – review & editing, writing – original draft, investigation, formal analysis. **John T. Echols:** Writing – review & editing, validation, software, methodology. **Amit R. Patel:** Writing – review & editing. **Matthew J. Wolf:** Writing – review & editing, supervision, investigation, funding acquisition. **Frederick H. Epstein:** Writing – review & editing, writing – original draft, supervision, funding acquisition, conceptualization.

## Declaration of Generative AI and AI-assisted technologies in the writing process

During the preparation of this work, the authors used ChatGPT in order to improve readability and clarity of the manuscript text. After using this tool, the authors reviewed and edited the content as needed and take full responsibility for the content of the publication.

## Declaration of competing interests

The authors declare the following financial interests/personal relationships which may be considered as potential competing interests: Frederick Epstein reports that financial support was provided by National Heart Lung and Blood Institute. Julia Bresticker reports that financial support was provided by American Heart Association Inc. Amit Patel is an associate editor of JCMR. If there are other authors, they declare that they have no known competing financial interests or personal relationships that could have appeared to influence the work reported in this paper.
