# Supplementary material for: Preclinical magnetic resonance imaging of proinflammatory epicardial adipose tissue: accelerated methods for simultaneous fatty acid composition and relaxation parameter mapping with relationships to tissue biomarkers
Source: J Cardiovasc Magn Reson. 2025 Aug 22;27(2):101947. doi: 10.1016/j.jocmr.2025.101947 (PMC12681544; doi:10.1016/j.jocmr.2025.101947)
Supplement: Supplementary file 1 — Supplementary material [file mmc1.docx]

| Cytokine Name | Abbreviation(s) |
| --- | --- |
| Granulocyte colony-stimulating factor | G-CSF |
| Eotaxin | CCL11 |
| Granulocyte-macrophage colony-stimulating factor | GM-CSF/CSF2 |
| Interferon-gamma | IFN-γ |
| Interleukin-1 alpha | IL-1α |
| Interleukin-1 beta | IL-1β |
| Interleukin-2 | IL-2 |
| Interleukin-3 | IL-3 |
| Interleukin-4 | IL-4 |
| Interleukin-5 | IL-5 |
| Interleukin-6 | IL-6 |
| Interleukin-7 | IL-7 |
| Interleukin-9 | IL-9 |
| Interleukin-10 | IL-10 |
| Interleukin-12 (p40 subunit) | IL-12 (p40) |
| Interleukin-12 (p70 subunit) | IL-12 (p70) |
| Interleukin-13 | IL-13 |
| Interleukin-15 | IL-15 |
| Interleukin-17 | IL-17 |
| Leukemia inhibitor factor | LIF |
| Lipopolysaccharide-induced CXC chemokine | LIX/CXCL5 |
| Interferon-gamma-induced protein 10 | IP-10/CXCL10 |
| Keratinocyte chemoattractant | KC/CXCL1 |
| Monocyte chemoattractant protein-1 | MCP-1/CCL2 |
| Macrophage inflammatory protein-1 alpha | MIP-1α/CCL3 |
| Macrophage inflammatory protein-1 beta | MIP-1β/CCL4 |
| Macrophage colony-stimulating factor | M-CSF/CSF-1 |
| Monocyte chemoattractant protein-2 | MIP-2/CXCL2 |
| Monokine induced by gamma interferon | MIG/CXCL9 |
| Regulated upon activation normal T-cell expressed and secreted | RANTES/CCL5 |
| Vascular endothelial growth factor | VEGF |
| Tumor necrosis factor alpha | TNF-α |

**Supplemental Table 1.** Cytokines included in the mouse 32-plex Luminex panel.
